# “Intrinsic” correlations and their temporal evolutions between winter-time PNA/EPW and winter drought in the west United States

**DOI:** 10.1038/srep19958

**Published:** 2016-01-27

**Authors:** Lin Piao, Zuntao Fu, Naiming Yuan

**Affiliations:** 1 Lab for Climate and Ocean-Atmosphere Studies, Dept. of Atmospheric and Oceanic Sciences, School of Physics, Peking University, Beijing, 100871, China; 2 Chinese Academy of Meteorological Science, Beijing, 100081, China

## Abstract

In this study, relations between winter-time Pacific-Northern America pattern (PNA)/East Pacific wave-train (EPW) and winter-time drought in the west United States over the period of 1951–2010 are analyzed. Considering traditional Pearson’s Correlation Coefficient can be influenced by non-stationarity and nonlinearity, a recently proposed method, Detrended Partial-Cross-Correlation Analysis (DPCCA) is applied. With DPCCA, we analyzed the “intrinsic” correlations between PNA/EPW and the winter drought with possible effects of ENSO and PDO removed. We found, i) significant negative correlations between PNA/EPW and drought on time scales of 5–6 years after removing the effects of ENSO, ii) and significant negative correlations between PNA/EPW and drought on time scales of 15–25 years after removing the effects of PDO. By further studying the temporal evolutions of the “intrinsic” correlations, we found on time scales of 5–6 years, the “intrinsic” correlations between PNA/EPW and drought can vary severely with time, but for most time, the correlations are negative. While on interdecadal (15–25 years) time scales, after the effects of PDO removed, unlike the relations between PNA and drought, the “intrinsic” correlations between EPW and drought takes nearly homogeneous-sign over the whole period, indicating a better model can be designed by using EPW.

Recently the west U.S. has experienced severe dryness, which presents a challenge to humans and the ecosystem there. The impact of soil moisture deficits can be acute, including enhanced wildfire risk^1^, damage to habitat of vulnerable species^2^, and land subsidence from excessive groundwater withdrawals^3^. As a result, scientists have been trying the best to figure out what has caused the severe drought. Most recent analysis and forecasting about west U.S. drought have focused on the Pacific Ocean to find reasons, specifically on oceanic patterns such as the El Ni*ñ*o Southern Oscillation (ENSO) and the Pacific Decadal Oscillation (PDO)^4^,^5^,^6^,^7^. For instance, it has been found that the La Ni*ñ*a events can bring dry conditions to the west United States, especially when PDO is in its negative phase, the teleconnections between La Ni*ñ*a and the west U.S. drought tend to be strong and stable. As a result, strong drought event may happen during La Ni*ñ*a events. However, few studies have paid close attention to the impact of atmospheric patterns on droughts, such as Pacific-Northern America pattern (PNA) and East Pacific wave-train (EPW). As we all know, PNA is one of the most prominent internal modes of low-frequency variability in the Northern Hemisphere extratropics. Its positive phase indicates above-average heights in the vicinity of Hawaii and the intermountain region of North America, while below-average heights over south of the Aleutian Islands and the southeastern United Sates. Different from PNA, the EPW is generated in the lower troposphere over the East Pacific and propagates predominantly eastward into North America and slightly upward, then eventually into stratosphere. Since the spatial and temporal variability of EPW is slightly different from that of PNA^8^,^9^,^10^, their influences may to some extent be different.

In this study, we focus on the influences of PNA and EPW on continental moisture balance which are quantified by self-calibrated Palmer Drought Severity Index (sc_PDSI) in the west U.S. on interannual and decadal-to-interdecadal time scales. These two timescales are the significant time scales for PNA and EPW which have been stated in Figs 1 and 2, see also^10^. Besides PNA and EPW, we also extract components of stationary planetary wave scale with zonal wavenumber from b = 1 to b = 3 derived from EPW, written as EPW_1*to*3_. EPW_1*to*3_ is discussed because motions of stationary planetary wave scale can reveal the impact of EPW on longer time scales^11^,^12^ noteworthily.

To study the possible influences of PNA and EPW on continental moisture balance, a straightforward way is to utilize the traditional Pearson’s correlation analysis. However, before analysis, we need to note that due to the effects of many nonlinear processes and external forcing in climate system, time series obtained in nature are usually characterized by multi-scaled structures and non-stationary^13^, and multi-series are normally linked via complex interactions. Therefore, the traditional Pearson’s correlation is not always appropriate and can provide erroneous results^14^ in some circumstances. For example, in^14^, the authors have argued that the calculated Pearson’s correlation coefficient of the summer rainfall over the middle-lower reaches of the Yangtze River (SRYR) and the previous winter-time (December, January and February) Ni*ñ*o3 Sea Surface Temperature Anomaly (Ni*ñ*o3-SSTA) is only 0.19 for the past 60 years (not statistically significant according to the student’s t-test), which is lower than expected since it is well acknowledged that SRYR are teleconnected with the previous winter-time East Pacific SSTA^15^. In this case, the traditional Pearson’s correlation seems to be insufficient. Considering ENSO has typical period of about 2 ~ 7 years, one may need to calculate better coefficients to reveal the correlations on multiple time scales, such as the DCCA cross-correlation coefficient^16^,^17^,^18^, which is based on the detrended cross-correlation analysis (DCCA)^19^, and can be used to quantify the levels of cross-correlations on different time scales^20^. However, as discussed in^14^, cross-correlations between two time series may also be altered by a common coupled third time series. Such as in our case, see Figs 1 and 2, PNA, EPW and EPW_1*to*3_ seem to be associated with ENSO and PDO simultaneously to some extent since the global wavelet spectrum analysis of the normalized indexes have shown that they have significant characteristic time scale of 3–5 years (ENSO typical period), while after 10 years low-pass filtering they have significant characteristic time scales of around 20 years (PDO typical period). Thus, it is necessary for us to capture the “intrinsic” correlations between two considered series on different time scales, with potential influences of other unconsidered series removed. In this study, we choose to apply a recently developed new method, detrended partial-cross-correlation analysis (DPCCA). DPCCA is based on DCCA and improved by including partial-correlation technique, therefore it is useful in moving the potential influences of unconsidered series, and reveal the so called “intrinsic” correlations between the two considered time series. In this work, we use this method to study the correlations between winter-time PNA/EPW and winter drought, with the possible influence of ENSO and PDO removed.

In addition, due to the non-stationarity in climate system, “intrinsic” correlations averaged over the whole time span can be different from instantaneous “intrinsic” correlations over specific time intervals, as discussed in^21^, where the temporal relationship between the North Atlantic Oscillation (NAO) and ENSO has been found variable over time. Therefore, we should also pay attention to the relations between atmospheric patterns and drought at different time intervals. In this case, besides applying DPCCA to the whole time span, we also do the same analysis to different time intervals. With the temporal evolution of “intrinsic” correlations calculated, we can better understand on what time scale and to what extent ENSO/PDO has influenced the “intrinsic” correlations between PNA/EPW and drought.

This paper is organized as follows. In the “Results” section, the “intrinsic” correlations and its temporal evolution between PNA/EPW and drought in the west U.S. are shown, and the potential causes are discussed. Then a brief conclusion and discussion are made in the following section. In the end of this paper, the data and the methods are described in detail.

## Results

### The “intrinsic” correlations between atmospheric patterns and west U.S. drought

In this study, we are interested in finding the correlations between atmospheric patterns PNA/EPW (characterized by Pacific-Northern America pattern index (PNAI)/East Pacific wave-train index (EPWI)) and the drought conditions in west United States. We mainly focus on the winter time since it has been recognized that the influences of the oceanic and atmospheric patterns are more significant during boreal winter^22^,^23^. We use the averaged sc_PDSI over west U.S. to characterize the moisture balance condition. The index is calculated according to station data and the detailed distribution of stations can be found in Fig. 3. Moreover, we characterize ENSO activity by using the Ni*ñ*o3.4 Sea Surface Temperature Anomaly (Ni*ñ*o3.4-SSTA) and PDO activity by using Pacific Decadal Oscillation index (PDOI).

In Fig. 4a–c, we analyze the correlations between PNAI/EPWI/EPWI_1*to*3_ and sc_PDSI by calculating DCCA cross-correlation coefficient *ρ*_*DCCA*_, which has been widely used^16^,^17^,^18^,^20^, with the influence of other series not been removed, see the black lines. In Fig. 4a, sc_PDSI is weakly correlated with PNAI on time scale of 5–8 years with cross-correlation coefficient *ρ*_*DCCA*_ close to −0.2 (not exceeding 90% confidence level). As for the cross-correlations between EPWI and sc_PDSI, see Fig. 4b, the cross-correlation coefficient *ρ*_*DCCA*_ is nearly of the same value (−0.25) on the time scale of 5 to 15 years (still not exceeding the 90% confidence level). But we could not say that there is no impact of PNA and EPW on moisture balance in west U.S., since the oceanic pattern such as the ENSO and PDO may play a role in the analysis. Therefore, we need to reveal the “intrinsic” correlations between PNAI and sc_PDSI, as well as the “intrinsic” correlations between EPWI/EPWI_1*to*3_ and sc_PDSI. After removing the influence of ENSO, whose typical period is 2–7 years, see the red solid line in Fig. 4a, significant negative cross-correlation coefficients *ρ*_*DPCCA*_ between PNAI and sc_PDSI are found over all time scales. On time scale of 5–6 years, the correlation even exceeds the 90% confidence level. This result is interesting, as we all know that the relations between ENSO and PNA is quite controversial. In^24^, the authors argued that ENSO cannot force PNA at all during warm ENSO events by analyzing ensemble general circulation model (GCM) simulations. However, later researches pointed out that PNA is triggered by tropical conventions, maybe the Madden-Julian Oscillation (MJO)^25^,^26^. In fact, it has been well accepted that ENSO SST forcing can selectively amplify natural forms of internal variability from the perspective of the background flow (the strength of the horizontal wind shear and the meridional potential vorticity gradient), then the forcing may influence the PNA to some extent^26^,^27^. Our results, from another perspective, proved the influence of ENSO on the PNA. As for the cross-correlations between EPWI and sc_PDSI, see the red solid line in Fig. 4b, they also exceeded the 90% confidence level on the time scale of 5 years after removing the influence of ENSO. Results discussed above indicate that ENSO has remarkable influence on its typical time scale (5–6 years), which may cover the cross-correlations between PNAI/EPWI and sc_PDSI. Furthermore, from Fig. 4a,b, the impact of ENSO seems to be stronger on PNA than on EPW at interannual time scale, and this may be due to the fact that PNA develops closer to the equator than EPW. Concerning EPWI_1*to*3_, which represents the impacts from motions of stationary planetary wave scale, we find that the cross-correlation coefficient *ρ*_*DCCA*_ becomes more negative at all time scale in comparison with the cross-correlation coefficient *ρ*_*DCCA*_ between EPWI and sc_PDSI, especially on the time scale of 4–6 years (exceeding the 90% confidence level), see Fig. 4c. This indicates that the motions of stationary planetary wave scale maybe the major factor that has influenced the winter-time drought in west U.S. Furthermore, after removing the influence of ENSO, see Fig. 4c, the cross-correlation coefficient *ρ*_*DPCCA*_ is only slightly different from *ρ*_*DCCA*_, which suggest the relations between EPWI_1*to*3_ and sc_PDSI in the west U.S. is only weakly influenced by ENSO.

In Fig. 4d–f, we focus on the correlations between atmospheric patterns and sc_PDSI on decadal to interdecadal time scales. Marked differences in the outputs between DPCCA (removing the influence of PDO) and DCCA are found over all time scales. Neither the cross-correlation coefficients *ρ*_*DCCA*_ between PNAI and sc_PDSI, nor the *ρ*_*DCCA*_ between EPWI and sc_PDSI can exceed the 90% confidence level on decadal to interdecadal (11–30 years) time scales. By comparison, the cross-correlation coefficient *ρ*_*DPCCA*_ between PNAI and sc_PDSI on time scale of 14–21 years (nearly −0.5) and *ρ*_*DPCCA*_ between EPWI and sc_PDSI on time scale of 15–24 years (nearly −0.5) are both above the 90% confidence level. When motions on stationary planetary wave scale are extracted from EPW, in Fig. 4f, the *ρ*_*DPCCA*_ between EPWI_1*to*3_ and sc_PDSI even exceeds the 90% confidence level over all time scales (11–30 years) and the cross-correlation can reach −0.6 on the time scale of 20 years. Therefore, as expected, oceanic patterns like ENSO and PDO indeed can mask the “intrinsic” correlations between atmospheric patterns and drought.

### Temporal evolutions of “intrinsic” correlations between atmospheric patterns and drought

Concerning the nonlinear character of climate system, the cross-correlation between two non-stationary series obtained from nature may not always be in line with the time-averaged value and it varies through time^21^. Therefore, in the following, we focus on the temporal evolutions of “intrinsic” correlations between PNAI/EPWI/EPWI_1*to*3_ and sc_PDSI.

To evaluate how the “intrinsic” correlations between PNAI/EPWI/EPWI_1*to*3_ and sc_PDSI in the west U.S. vary through time, we calculate *ρ*_*DCCA*_ (for the purpose of comparison) and *ρ*_*DPCCA*_ within sliding windows with sizes ranging from 5 to 30 years, and different window sizes represent different time scales. Then, we acquire the plots in Fig. 5.

In Fig. 5a,d,g, on the time scale of 5–6 years, the values of cross-correlations coefficient *ρ*_*DCCA*_ between PNAI/EPWI/EPWI_1*to*3_ and sc_PDSI varies from positive to negative or from negative to positive over time intermittently. This result is in line with our concerns above, that the correlations between atmospheric pattern and drought are not always stationary through time, and the sign of correlation coefficient may switch frequently from positive to negative over the selected intervals. The positive correlations found in DCCA may be due to the influence of ocean pattern (ENSO) on interannual time scale, which is so strong that may mask the “original influence” (the negative cross-correlation). In order to confirm the hypothesis above and to understand when and to what extent that ENAO has influenced the “intrinsic” correlations between atmospheric patterns and drought, see Fig. 5b,e,h, we applied DPCCA. After removing the impact of ENSO, more negative cross-correlation coefficients *ρ*_*DPCCA*_ are found during the period when the *ρ*_*DCCA*_ are also negative, see Fig. 5a/d/g on the time scale of 5–6 years. Especially for the results between PNAI and sc_PDSI, which is in line with our findings in Fig. 4, that the influence of ENSO seems to be greater on the “intrinsic” correlations between PNAI and sc_PDSI.

The temporal evolutions of “intrinsic” correlations between atmospheric patterns and drought on the decadal-to-interdecadal time scale are revealed in Fig. 5c,f,i. On time scale of 15–25 years, significant differences between the results of DCCA and DPCCA are found. Nearly all positive values during the last half period in Fig. 5a,d,g disappear in Fig. 5c,f,i, which confirms the significant, negative, time-averaged cross-correlation coefficient *ρ*_*DPCCA*_ we found in Fig. 4d,e,f. As for the temporal evolutions of the “intrinsic” correlations between PNAI and sc_PDSI, the values are negative during the period of 1960–1985, but close to 0 during the period of 1985–2000. In contrast, when it comes to the temporal evolutions of the “intrinsic” cross-correlations between EPWI/EPWI_1*to*3_ and sc_PDSI, we find nearly homogeneous-signed, negative values over the whole study period. This result makes EPWI/EPWI_1*to*3_ better potential predictors for building models to forecast dry-wet conditions in the west U.S. on the time scale of 15–25 years.

To sum up, from Fig. 5 we can clearly figure out on what scale and to what extent that ENSO/PDO can modulate the “intrinsic” correlations between atmospheric patterns and drought on the interannual/decadal-to-interdecadal time scale over time. After removing the influence of ENSO, both PNA and EPW can negatively contribute to the west U. S. drought on interannual time scale, but not invariant through time. While after removing the influence of PDO, to our surprise, EPW can negatively contribute to the west U. S. drought during the whole study period on decadal-to-interdecadal time scale.

## Conclusion and Discussion

**Figure 1:**
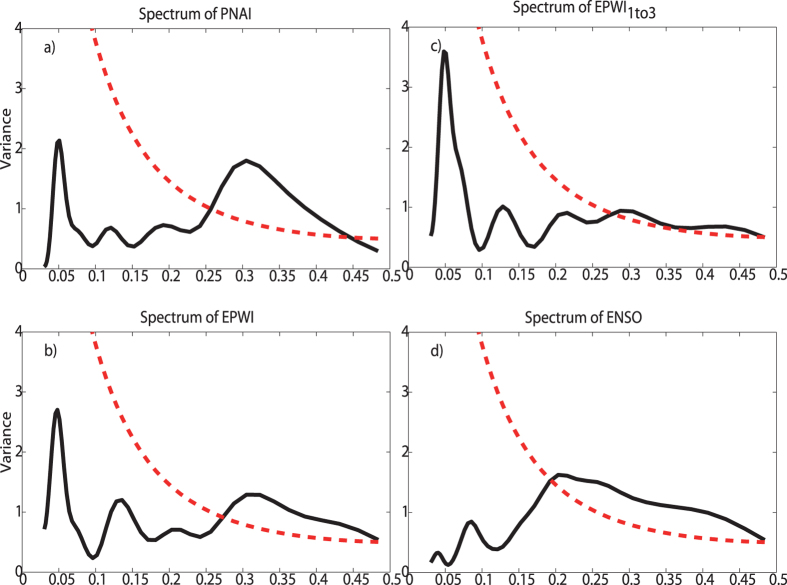
Global wavelet spectrum of normalized atmospheric patterns and ENSO (black & solid). (**a**) is the result of PNAI, (**b**) is the result of EPWI, (**c**) is the result of EPWI_1*to*3_, and (**d**) is the result of Ni*ñ*o3.4-SSTA. The red dashed lines are the 95% confidence spectrum for the red-noise AR (1) process with lag-1 of 0.5.

**Figure 2:**
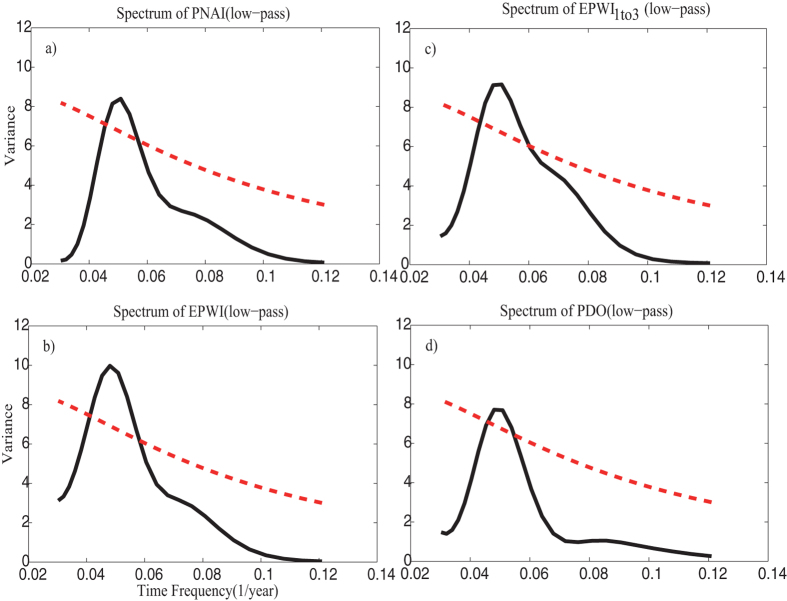
Global wavelet spectrum of 10-years low-pass filtered normalized atmospheric patterns and PDO (black & solid). (**a**) is the result of PNAI, (**b**) is the result of EPWI, (**c**) is the result of EPWI_1*to*3_, and (**d**) is the result of PDOI. The red dashed lines are the 95% confidence spectrum for the red-noise AR (1) process with lag-1 of 0.5.

In this paper, winter drought (sc_PDSI) in the west United States over the past 60 years are studied. Different from recent researches, where the influence of oceanic patterns (ENSO and PDO) are widely studied, we in this study mainly focus on the effects of atmospheric patterns (PNA/EPW).Considering both the atmospheric patterns (PNA/EPW) and the west U.S. drought may be commonly influenced by ENSO and PDO (see Figs 1 and 2), a recently developed method, DPCCA, is applied in this study. With DPCCA, we revealed, for the first time, the “intrinsic” correlations between PNA/EPW and west U.S. drought on different time scales with possible influences of oceanic patterns (ENSO and PDO) removed. To illustrate the possible influences of ENSO and PDO, we also calculated the DCCA cross-correlation coefficient *ρ*_*DCCA*_ for comparison, which can only be applied to two time series, without removing the influence of other common coupled time series. From *ρ*_*DCCA*_, we find that the atmospheric patterns PNA/EPW indeed have some correlations with sc_PDSI in the west U.S., but neither of them can exceed the 90% confidence level. Only when we study the relations between EPWI_1*to*3_ and sc_PDSI, significant correlations exceeding 90% confidence level can be found on time scale of 4–6 years. However, if we apply DPCCA, significant negative correlations between PNA/EPW and the winter drought are found on interannual (5–6 years) time scales after removing the influence of ENSO, and significant negative correlations between PNA/EPW and the winter drought are found on time scales of 15–25 years after removing the impact of PDO. Furthermore, the temporal evolutions of the “intrinsic” correlations are also investigated, which reveal that the relations between the atmospheric patterns and the winter drought in west U.S. cannot always keep unchanged over time. On time scales of 5–6 years, after removing the effects of ENSO, the “intrinsic” correlations between PNA/EPW and drought can vary severely with time, but for most time, the correlations are negative. While on decadal-to-interdecadal (15–25) years, we are surprised to find that the cross-correlation coefficients *ρ*_*DPCCA*_ tend to become consistently negative over the whole time span after removing the impact of PDO, especially for the *ρ*_*DPCCA*_ between EPWI/EPWI_1*to*3_ and sc_PDSI. This indicates EPWI/EPWI_1*to*3_ may be better predictors for building models to forecast dry-wet conditions in the west U.S.

The “intrinsic” correlations and their temporal variations we found in this study proved that there are indeed close relations between PNA/EPW and the winter drought in west U.S., but we need to focus on specific time scales. In fact, the correlations we found can be explained from previous studies. For the negative correlations between PNAI and sc_PDSI, it has been widely accepted that the positive phase of PNA pattern is associated with above-average air temperatures over the west U.S., which will result in severe drought later (quantified by lower sc_PDSI). Concerning the “intrinsic” negative correlation between EPWI and sc_PDSI in the west U.S., we can explore it from the aspect of precipitation. In fact, it has been well acknowledged that precipitation deficit is a prerequisite for the moisture deficit, which will further lead to drought. In west U.S. the most important part of precipitation is winter/spring snow. When the active EPW happens, there will be recurring pole-ward deflection of the cool-season storm track forced by a region with persistent high atmospheric pressure, which steers Pacific storms away from the west U.S. and then leads to precipitation deficits^7^,^28^,^29^. As for the question of why ENSO/PDO has so remarkable influence on the “intrinsic” correlations between PNAI/EPWI/EPWI_1*to*3_ and sc_PDSI, we may explain it as following: the El-Ni*ñ*o-related dry-wet changes can result in wetter conditions (quantified by larger sc_PDSI) at the southwest U.S., while La Ni*ñ*a-related dry-wet changes can lead to drier conditions there. Moreover, when ENSO is in phase with PDO, the ENSO-induced dry-wet changes are magnified^7^,^30^,^31^. Therefore, after removing the oceanic variability, we get magnified negative cross-correlations between atmospheric patterns and drought on the interannual (5–6years) and decadal-to-interdecadal (15–25 years) time scales.

Furthermore, we need to note that in this study we only take the influence of conventional El Ni*ñ*o (Eastern Pacific El Ni*ñ*o, EP-El Ni*ñ*o) into account for the interannual time scale. It has been widely acknowledged that El Ni*ñ*o Modoki (Central Pacific El Ni*ñ*o, CP-El Ni*ñ*o) appeared more frequently in recent years and its pattern is distinct from the pattern of the conventional El Ni*ñ*o^32^,^33^. Therefore, it should be helpful to include El Ni*ñ*o Modoki into our analysis. Besides, on decadal time scale, other factors such as the land surface processes may also need to be taken into account since it can feed back positively on the moisture balance. Therefore, more detailed analysis is still needed in future.

## Data and Methods

### Data

The data used in this study are Pacific-Northern America pattern index (PNAI) and East Pacific wave-train index (EPWI) both representing atmospheric patterns. The PNAI which reflects a quadripole pattern of 500-millibar height anomalies is downloaded from the National Oceanic & Atmospheric Administration (NOAA) (http://www.esrl.noaa.gov/psd/data/climateindexes/). And the EPWI is defined as the volume-averaged vertical stationary wave fluxes in the domain (30°–60°*N*, 170°–120°*W*; 925–500*hPa*) using the three-dimensional wave fluxes proposed by^34^, the function is presented as below:


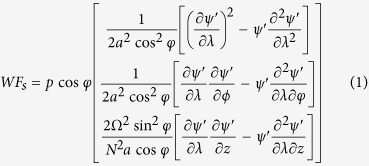


where the vector *WF*_*s*_ is the three-dimensional wave flux of the stationary waves, which is derived under the quasi-geostrophic approximation. In this function, *p* = *pressure*/1000 hPa, *z* = −*H*ln*p* and H is a constant scale height (H is set equal to 7.5 km for all computations here). *a*, Ω, *φ*, *λ* represent the Earth’s radius, the Earth’s rotation rate, latitude and longitude, respectively. *ψ*′ is the small perturbation of the streamfunction to its zonal mean. *N* is the buoyancy frequency. The EPWI is calculated by using the monthly mean gridded reanalysis data provided by the National Centers for Environmental Prediction (NCEP) and the National Center for Atmospheric Research (NCAR). More than that, we also extract components on stationary planetary wave scales with zonal wavenumber *b* = 1 to *b* = 3 derived from EPW and the stationary planetary wave scale index of EPW is written as EPWI_1*to*3_. EPWI_1*to*3_ is also discussed here because motions on stationary planetary wave scales can show the impact of EPW on longer time scales remarkably^11^,^12^.

We characterize ENSO activity using Ni*ñ*o3.4 Sea Surface Temperature Anomaly (Ni*ñ*o3.4-SSTA) and PDO activity using Pacific Decadal Oscillation index (PDOI). The Ni*ñ*o3.4-SSTA data is obtained from the website of KNMI Climate Explorer. The PDOI is acquired from the NOAA.

**Figure 3:**
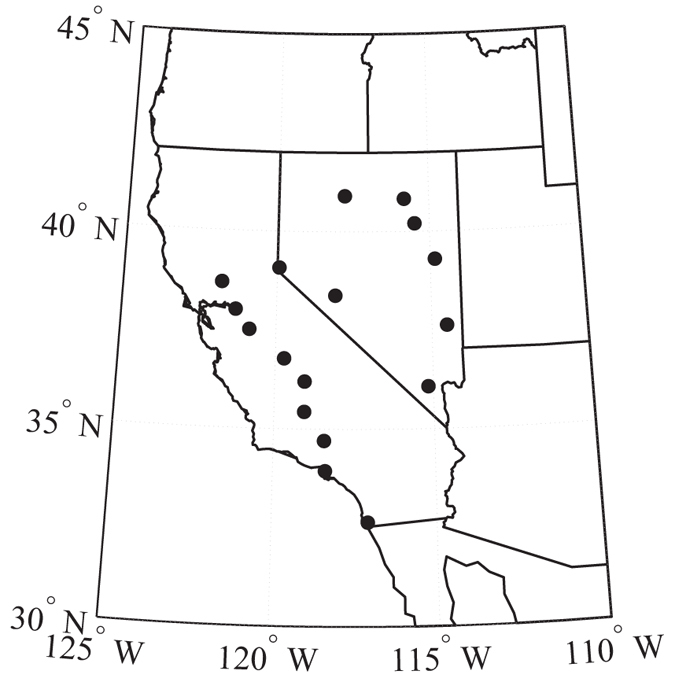
Geographic locations of the stations in the west U.S. 17 stations in the west U.S. are used, including 9 stations in California and 8 stations in Nevada. Their locations are shown as the solid circles. We generate the figure by using Matlab.

**Figure 4:**
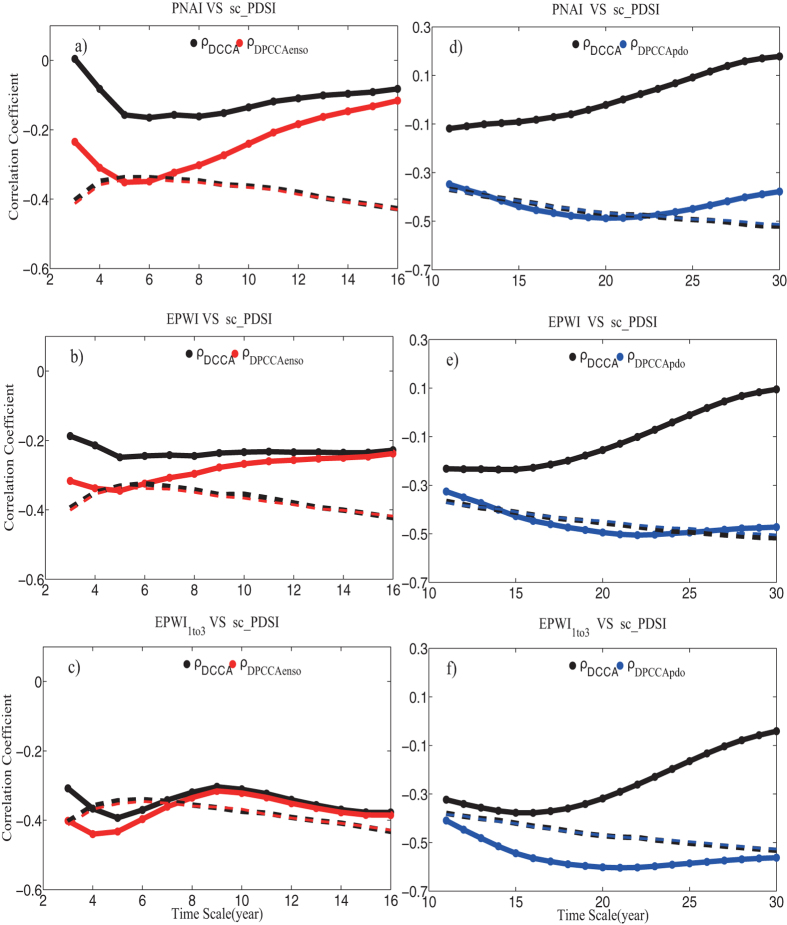
Correlations between atmospheric patterns and sc_PDSI. DCCA cross-correlation coefficients *ρ*_*DCCA*_ are shown in black color, while DPCCA coefficients *ρ*_*DPCCA*_ are shown in red. Results between PNAI and sc_*PDSI* are shown in (**a**)/(**d**), between EPWI and sc_PDSI are shown in (**b**)/(**e**), and between EPWI_1*to*3_ and sc_PDSI are shown (**c**)/(**f**). On the left hand side, we calculate *ρ*_*DPCCA*_ by removing the effects of ENSO, while on the right hand side, *ρ*_*DPCCA*_ is determined with the effects of PDO removed. The black dashed line represents the 90% significance criterions of *ρ*_*DCCA*_ on different time scales, while the red dashed line represents the criterions of *ρ*_*DPCCA*_. (They are both obtained from Monte-Carlo Simulations. We shuffled the considered time series and repeated the DCCA/DPCCA calculations for 5000 times. The top 5% smallest values (on different time scales) are given in the dashed lines.)

**Figure 5:**
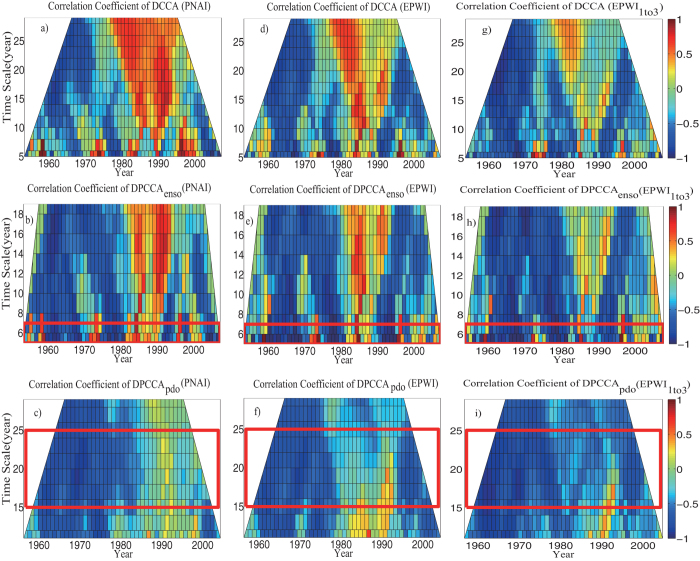
Temporal evolutions of the correlations between atmospheric patterns and sc_PDSI. Outputs of DCCA are shown in the first row (**a**,**d**,**g**); outputs of DPCCA (with the influence of ENSO removed) are shown in the second row (**b**,**e**,**h**); outputs of DPCCA (with the influence of PDO removed) are shown in the bottom row (**c**,**f**,**i**). The left column shows the results between PNAI and sc_PDSI (**a**–**c**), the middle column shows the results between EPWI and sc_PDSI (**d**–**f**), while the right column shows the results between EPWI_1*to*3_ and sc_PDSI (**g**–**i**).

The drought index used here is sc_PDSI downloaded from (http://droughtatlas.unl.edu/Data.aspx). The sc_PDSI is based on Palmer Drought Severity Index (PDSI) which is calculated from a water-balance model that is forced by observed precipitation and temperature and is closely related to precipitation and soil moisture content; the PDSI has been widely used to study aridity changes. To improve the spatial comparability, sc_PDSI is proposed, which calibrates the PDSI using local, instead of using the fixed coefficients^35^. And the average sc_PDSI over the west U.S. is calculated according to 17 station data, see Fig. 3.

Owing to the length of available data, in this paper we only focus on the time period of 1951–2010, with only winter-time (December, January and February) data selected. Because the influences of the oceanic and atmospheric patterns in the Pacific ocean on climate are much significant in the boreal winter^22^,^23^. In addition, winter precipitation is an important predictor of annual tree growth, particularly in the west U.S., and the drought include persistence from prior winter^30^. All indexes are normalized to have unit variance.

### Methods

In this subsection, we will explain DPCCA briefly.

Consider *m* time series of length *L*, 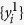, 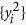, ···, 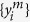, (*i* = 1, 2, 3, *L*). Firstly, the random walk profile is calculated by integrating the series, and the equation is stated as below:


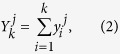


where *j* = 1, 2, 3, *m*, *k* = 1, 2, 3,, *L*. Secondly, each profile is divided into *L* − *s* overlapping boxes. Each box contains *s* + 1 values. For box that starts at *i* and ends at *i* + *s*, we define the local trend 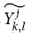 by using a polynomial fit. Then, we get the detrended walk as the difference between the original walk and the local trend, as:


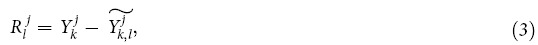


where *l* = 1, 2, 3, ···, (*L* − *s*)(*s* + 1). By calculating the covariance between any two residuals, we get,


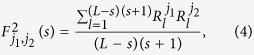


where *j*_1_, *j*_2_ = 1, 2, 3, *m*. Then we can get a *m* × *m* covariance matrix,


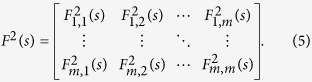


In the next step, the cross-correlation coefficients between any two time series are obtained according to the procedure proposed by^16^,^18^,


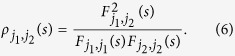


and a *m* × *m* matrix can again be obtained,


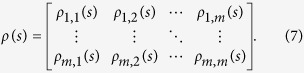


where each component in the above matrix ranges from −1 to +1 and represents the level of cross-correlation on time scale of *s*. If we only consider two time series (*m* = 2), *ρ*_1,2_(*s*) is the traditional DCCA cross-correlation coefficient *ρ*_*DCCA*_(*s*). However, we should note that this matrix only provides correlation information of two time series without removing the influence of other coupled series. In order to remove the possible influence of other series, partial-correlation method is used as following.

One first calculate the inverse matrix of *ρ*(*s*),


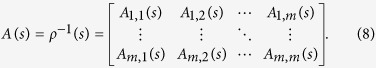


Then the partial-cross-correlation between two time series 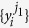 and 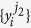 can be obtained as,


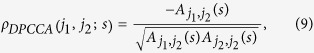


The coefficient *ρ*_*DPCCA*_(*j*_1_, *j*_2_; *s*) can be used to characterize the “intrinsic” correlations between two time series on time scale of s, with possible influence of other series 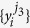, 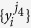, ···, 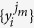 removed^14^. By changing *s*, we can further get the partial-cross-correlation coefficients between two time series on different time scales.

## Additional Information

**How to cite this article**: Piao, L. *et al.* “Intrinsic” correlations and their temporal evolutions between winter-time PNA/EPW and winter drought in the west United States. *Sci. Rep.* **6**, 19958; doi: 10.1038/srep19958 (2016).
